# A Phage Display-Identified Short Peptide Capable of Hydrolyzing Calcium Pyrophosphate Crystals—The Etiological Factor of Chondrocalcinosis

**DOI:** 10.3390/molecules26195777

**Published:** 2021-09-24

**Authors:** Radosław W. Piast, Rafał M. Wieczorek, Nicola Marzec, Maciej Garstka, Aleksandra Misicka

**Affiliations:** 1 Faculty of Chemistry, University of Warsaw, Pasteura 1, 02-093 Warsaw, Poland; wieczorek@chem.uw.edu.pl; 2 Inter-Faculty Individual Studies in Mathematics and Natural Sciences, University of Warsaw, Stefana Banacha 2C, 02-087 Warsaw, Poland; n.marzec@student.uw.edu.pl; 3 Department of Metabolic Regulation, Faculty of Biology, University of Warsaw, Miecznikowa 1, 02-096 Warsaw, Poland; garstka@biol.uw.edu.pl

**Keywords:** chondrocalcinosis, CPPD, pseudogout, phage display, peptides

## Abstract

Chondrocalcinosis is a metabolic disease caused by the presence of calcium pyrophosphate dihydrate crystals in the synovial fluid. The goal of our endeavor was to find out whether short peptides could be used as a dissolving factor for such crystals. In order to identify peptides able to dissolve crystals of calcium pyrophosphate, we screened through a random library of peptides using a phage display. The first screening was designed to select phages able to bind the acidic part of alendronic acid (pyrophosphate analog). The second was a catalytic assay in the presence of crystals. The best-performing peptides were subsequently chemically synthesized and rechecked for catalytic properties. One peptide, named R25, turned out to possess some hydrolytic activity toward crystals. Its catalysis is Mg^2+^-dependent and also works against soluble species of pyrophosphate.

## 1. Introduction

Calcium pyrophosphate deposition (CPPD) is a metabolic disease primarily affecting the elderly population, and it is characterized by the deposition of calcium pyrophosphate (CaPP) crystals in synovial fluid. The presence of crystals may not result in symptoms, but be present as an incidental finding of chondrocalcinosis (CC) during X-ray imaging, or present as painless lumps. However, they can also contribute to acute CaPP crystal arthritis or chronic arthropathy with structural changes in osteoarthritis [1,2]. Historically, because of the similar symptoms, CC was confused with gout. It was only distinguished by McCarty et al. in 1962 as a separate disorder, and a new name was proposed: pseudogout [3]. CPPD is common, and the occurrence is correlated with age [4]. In the UK, prevalence was approximately 10–15% in a community sample of 65–75 year-olds; and above 40% of the over-80 population suffer from CPPD associated symptoms [5,6,7]. It had a prevalence of 0.42% in an Italian population survey, making it the fourth most common musculoskeletal condition [8].

There are 12 known crystallographic forms of CaPP crystals, but only monoclinic and triclinic forms are found in vivo. In vitro crystallization studies have shown that initial deposits of CaPP are amorphous or orthorhombic, and that a triclinic form is the final form of interconversion among different crystal forms [9].

Treatment for chondrocalcinosis is done in response to symptoms. An acute form is treated with rest, in situ application of ice, joint aspiration, colchicine and intra-articular corticosteroid injections [10], immunosuppressive agents [11,12], and surgical joint replacement in severe cases of arthropathy [11,13]. Attempts to shed CaPP crystals with disodium EDTA and Mg^2+^—potent in vitro solubilizers—were a therapeutic failure [14]. Some dissolution of CaPP crystal in vitro has been achieved with the use of polyphosphates and by the combined activity of alkaline phosphatase and inorganic pyrophosphatase [15,16]; however, the enzymes turned out to be unsuccessful in vivo and are not currently used in the treatment of CPPD [17]. Therefore, at present, there are no treatments to safely eliminate CaPP crystals once deposited [10].

Nonenzymatic hydrolysis of pyrophosphate occurs at one of the lowest rates found in nature. This is caused by the presence of many canonical structures of that molecule that shield the central phosphorus atom from the eventual nucleophilic attack. In biological systems, a pyrophosphate is hydrolyzed by inorganic pyrophosphatase and other enzymes performing phosphoryl transfer reactions. To polarize the phosphate moiety, making the central phosphorus atom more electrophilic and more vulnerable to nucleophilic attack, these enzymes require the presence of a divalent cation, such as Mg^2+^ or Mn^2+^ [18,19].

Here, we are describing an attempt to find a peptide able to dissolve/hydrolyze CaPP from a random phage-display-delivered library. The method included two selection steps based on the affinities and catalytic properties of bacteriophages and subsequent testing of chemically synthesized peptides based on their phage-displayed counterparts. Activity assays were mostly based on ion chromatography of left-over crystals. We also studied the impacts of Mg^2+^ and Ca^2+^ on the reaction.

## 2. Results and Discussion

### 2.1. Phage Display

Using alendronate-functionalized resin and a high concentration of EDTA as selective factors for M13 bacteriophages, we isolated 19 viable phages from the PhD-C7C (called S01 to S19) and 28 from the PhD-12 library (R01 to R28). All of them were tested for possessing hydrolytic properties.

### 2.2. Catalytic Assay for Bacteriophages

**Figure 1:**
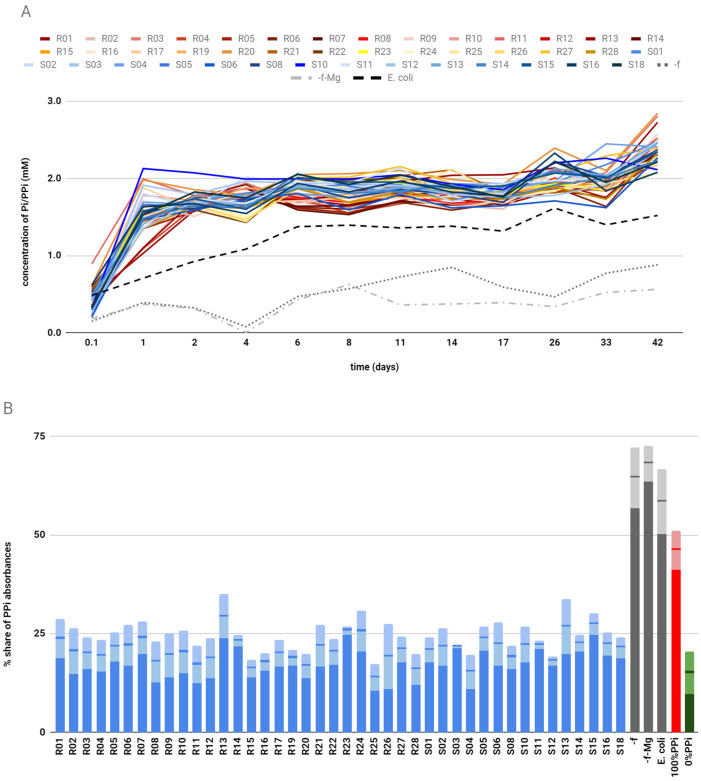
(**A**) The monitoring of the Pi/PPi levels in the supernatant during the catalytic selection of bacteriophages. The error is the standard deviation of a sample with a known concentration. The standard deviation was equal to 0.11 mM but is omitted for clarity; bacteriophages from PhD-12 library are shown in warm colors and from PhD-C7C in cold colors. Blind probes: without bacteriophage [-f], without bacteriophage and Mg^2+^ ions [-f-Mg] and with a sample isolated from noninfected with M13 bacterial host [*E. coli*] are shown in gray dotted lines. (**B**) Percentages of absorbance from fractions eluting pyrophosphate after ion chromatography. The sum of all absorbance fractions (phosphates and pyrophosphates) is 100. Bacteriophages tested in this research are shown in blue. Blind probes: without bacteriophage [-f], without bacteriophage and Mg^2+^ ions [-f-Mg] and with a sample isolated from noninfected with M13 bacterial host [*E. coli*] are shown in gray. Control samples with calcium pyrophosphate crystals [100%PPi] are shown in red, and calcium phosphate in green [0%PPi]. Error bars, in dull colors on the tops of the bars, represent standard deviations.

All the bacteriophages tested raised the level of Pi/PPi in the supernatant (Figure 1A). The concentration was 2–3 times higher in comparison to the blind probe where water was used instead of phages (-f); 3-5 times higher in comparison to the blind probe with no phages and no Mg^2+^ (-f-M); and up to twice as high in comparison to the blind probe (*E. coli*).

The divalent cations level was significantly higher in the blind probes, especially in (-f). The presence of phages, in general, decreased the amount of Mg^2+^/Ca^2+^ in the solution.

Results from ion chromatography are shown as percentages of absorbance from fractions eluting PPi to the sum of Pi and PPi absorbances. Each final value is a median of all three results from three separate experiments (Figure 1B).

We have chosen peptides for sequencing depending on the results of this experiment. The selection criterion was outstanding behavior in any of the tested properties—for instance, the lowest divalent cation concentration or the fastest initial rise in phosphate concentration. We have sequenced 15 peptides.

During the sequencing, a new type of library was detected. PhD-12 delivered linker-truncated form with the architecture N’-X12-S-pIII-C’ with the GGG fragment deletion. We have named this form PhD-12-G3.

Since the concentration of divalent cations, phosphate monitoring, and PPi/Pi percentage did not give us a clear picture of what potential hydrolysis may look like, our main criteria of choice for the synthesis of peptides became the PPi/Pi percentage and rational sequence analysis. Peptides R28, R25, and S04 were chosen, since they had the lowest PPi/Pi percentages. Peptides R15 and S10 were chosen because they had residues commonly associated with phosphate transfer active sides: negatively charged acidic Glu and Asp for divalent cation coordination, and positively charged Arg and His for pyrophosphate coordination (Table 1) [20,21].

### 2.3. Catalytic Assay for Peptides

During 8 days of incubation, we monitored levels of PPi and divalent cations in the supernatant after centrifugation, but those results were inconclusive (Figure 1A).

**Figure 2:**
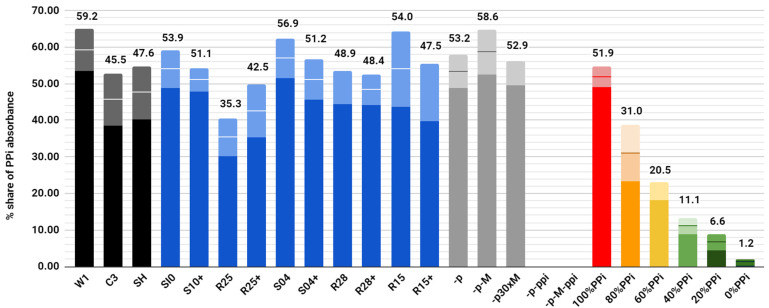
Percentage shares of absorbance from fractions eluting pyrophosphate. The sum of all absorbance fractions (phosphates and pyrophosphates) is 100. Control peptides are shown as black colums. Peptides (basic and elongated forms) studied in this research are shown in blue. Blind samples are shown in grey and correspond to: lack of peptide [-p], lack of peptide and Mg^2+^ ions, lack of peptide and 30 times higher Mg^2+^ ions concentration [-p30xM], lack of peptide and CaPP crystals [-p-ppi], lack of peptide, Mg^2+^ ions and CaPP crystals [-p-M-ppi]. The last 5 columns form a standard curve with a percentage of pirophosphate [PPi] calcium (red column) to phosphate calcium (dark green column) as indicated in the name of the sample. Error bars, in dull colors on the tops of the bars, represent standard deviations.

Ion chromatography showed that from all the peptides tested, only R25 and R25+ showed any hydrolytic activity toward CaPP crystals in comparison to the blind probe, hydrolyzing 18% and 10% of the pyrophosphate, respectively, according to the standard curve (Figure 2).

**Figure 3:**
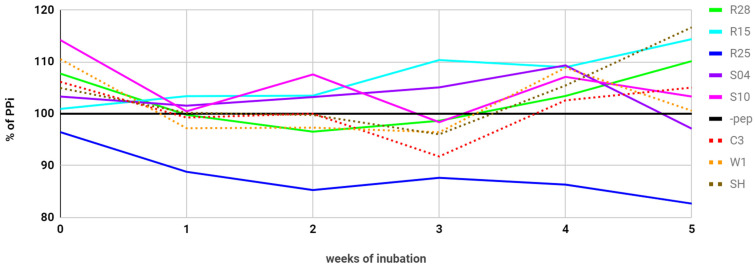
Levels of PPi during 5 weeks of incubation of peptides studied and peptides used as controls with calcium pyrophosphate crystals and magnesium ions in the alkaline environment. The addition of extra Mg^2+^ was done after the measurements in the fourth week of the experiment (not shown on the graph). The error is the standard deviation of a sample with a known concentration. It was equal to 3.27% but omitted from the graph for clarity.

To learn how long it would take for the R25 peptide to hydrolyze a whole sample of CaPP, we conducted another experiment where we were testing the percentage of PPi hydrolyzed over 5 weeks. Unfortunately, it turned out that the curve of hydrolysis flattens when approximately 85% PPi is left over, suggesting that the reaction can be inhibited by calcium released from the crystal. Calcium is a known inhibitor of phosphate transfer reactions. Alternatively, peptide might only hydrolyze some specific fragments of the crystal. To eliminate the first option, we added MgCl_2_ to the reaction a week before the last measurements—enough to increase the concentrations of pyrophosphate and Mg^2+^ (to 50 mM)—but no further drop in the PPi amount was observed (Figure 3).

**Figure 4:**
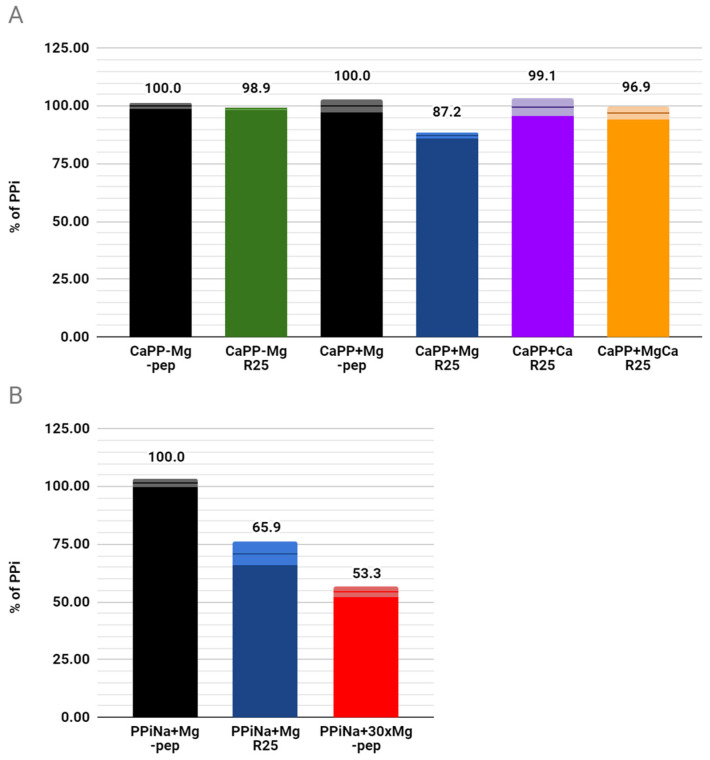
(**A**) Determination of Mg^2+^ and Ca^2+^ dependency of R25 in the hydrolysis of CaPP crystals; (**B**) Hydrolytic properties of R25 and a high concentration of Mg^2+^ against a soluble species of pyrophosphate—sodium pyrophosphate (PPiNa). Samples with Mg^2+^, Ca^2+^ ions or both indicated as +Mg, +Ca and +MgCa respectively. Samples without peptide R25 marked as -pep. Error bars represent standard deviations of 3 separate results, and are shown as dim sections at the tops of the bars.

To determine if the presence of Mg^2+^ is necessary for the catalysis, we have performed an experiment in which we incubated a reaction cocktail with R25, CaPP crystals, and buffer, but without Mg^2+^. After 7 days of the experiment, we have assessed the percentage share of PPi absorbance after ion chromatography in the pellet. Then we have added Mg^2+^ and incubated the reaction for another 7 days. After that, we measured the percentage share of the pyrophosphate absorbance after ion chromatography again. The experiment was repeated three times. Results showed that after incubation without Mg^2+^, levels of pyrophosphate remained high, with a median of 98.9%, in comparison to blind samples. After the addition of Mg^2+^ however, the percentage share of PPi dropped to 87.2% in comparison to blind samples (Figure 4A). We repeated the experiment with the addition of Ca^2+^ and Ca^2+^/Mg^2+^ (100 mM each) to learn the impact calcium ions have on the reaction. Both samples showed no or minimal level of hydrolysis, suggesting strong inhibition of the reaction by Ca^2+^ ions (Figure 4A).

In addition, we decided to determine whether the R25 peptide has hydrolytic activity toward soluble pyrophosphate species. For this purpose, we prepared a separate experiment substituting CaPP crystals with 5 mM Na_4_P_2_O7·10H_2_O and incubated the reaction cocktail for 14 days. Samples with R25 showed a decrease of the amount of the pyrophosphate left, by approximately 56%, in comparison to blind samples (Figure 4B). We also compared this result with a 30 times higher concentration of Mg^2+^ known to raise the rate of hydrolysis of pyrophosphate [14]. Unlike in experiments with CaPP, Mg^2+^ increased the hydrolysis of soluble pyrophosphate. In our conditions, after 14 days, Mg^2+^ catalyzed hydrolysis was nearly 14% more efficient than R25 catalyzed hydrolysis. All results are presented as medians of three independent experiments. All the percentage assessments were performed by comparing percentage shares of PPi absorbances from the standard curve.

Knowing that R25 is being inhibited by Ca^2+^ ions, we calculated the approximated total turnover number (TTN) defined as the number of molecules of substrate that a catalyst can convert before becoming inactivated [22]. For R25, this number was 129 ± 6 (where the error is the standard deviation between results of repeated experiments), meaning that approximately 129 molecules of PPi were hydrolyzed by one R25 molecule before the catalyst was inactivated.

### 2.4. Molecular Dynamics

**Figure 5:**
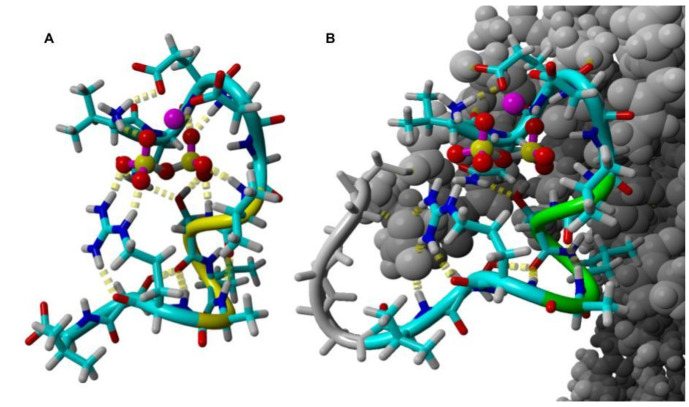
Plausible structures of R25 as a peptide alone (**A**) and joined to a pIII coat protein via a linker (**B**). The peptide is shown as sticks colored by atoms, pyrophosphate as sticks and balls, and Mg^2+^ is a pink sphere. pIII protein and the linker are shown in gray.

In order to learn how R25 works, we analyzed the molecular dynamics of this peptide attached to the pIII coat protein, and R25 alone. Although we found few potential structures for stand-alone R25 peptide, the ones structurally close to the one obtained with pIII protein had the lowest energy. Therefore, we acknowledged it as a plausibly native and active structure of the R25 peptide. (Figure 5). According to this structure, R25 binds tightly to a pyrophosphate molecule via Arg10, the N-terminal amine group, and the nest structure formed by the backbone of the QEDGV motif. Mg^2+^ is coordinated monodentaly by side chains of Glu3 and Asp4. The pIII protein seems to only stabilize the R25 via hydrophobic interactions. The R25 peptide deprived of the support of pIII and exposed to molecular dynamics remained stable, and its structure remainder relatively unchanged even after 50 ns of simulation (RMSD of 1.5 Å between the molecule simulated and the wild type docked to the pIII protein). Since the peptide lacks conspicuous catalytic residues able to act with a pyrophosphatase-like mechanism of providing a hydroxyl ion to nucleophilically attack the central atom of phosphorus of pyrophosphate molecule [23], the most probable mechanism of the reaction is one in which hydrolysis is initialized by the hydroxyl ion provided from the alkaline environment of the reaction cocktail, which explains the slow reaction rate.

### 2.5. Peptide Affinity Assay

**Figure 6:**
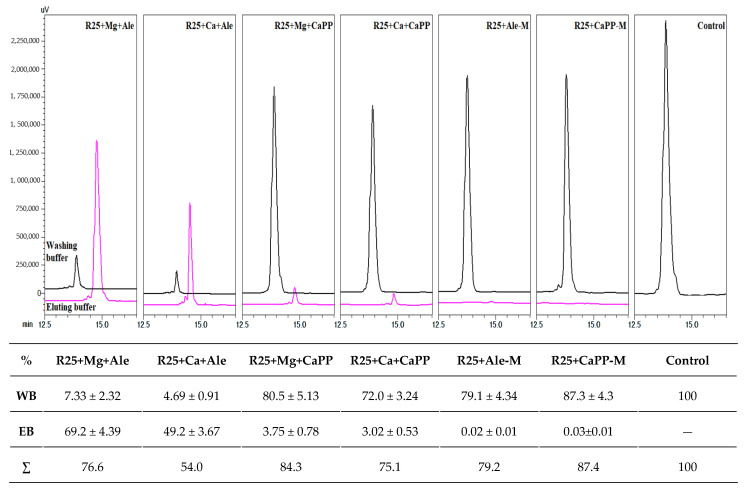
R25 affinity assay using alendronate-functionalized resin (Ale) and CaPP crystals in the presence of Mg^2+^ [+Mg] and Ca^2+^ [+Ca] ions. [−M] indicates a lack of divalent cations. HPLC chromatograms for R25 eluted in washing buffer (WB) and eluting buffer (EB) fractions, shown as percentages in comparison to the control (by surface areas of the peaks). Relative absorbance shown in μV and retention time in minutes. Chromatograms for eluted buffer fractions were shifted along both x and y axes for better visualization. Each error is the standard deviation of 3 measurements.

As was expected, the peptide did bind to the alendronic acid resin in the presence of divalent cations and did not bind when such cations were absent. In contrast, very small amounts of R25 bonded to CaPP crystals in the presence of ions, and practically no bonding occurred without cations. This could be because R25 dissolves the crystal without binding to it, as binding requires wrapping the peptide’s main chain around the pyrophosphate/alendronic acid molecule, which is impossible in the case of the crystal without dissolving it. Worth mentioning is the fact that sums of R25 in the washing fractions and eluted fractions in samples with Ca^2+^ ions and alendronic acid were not as large as those in other samples. The former lost approximately 15–25% of R25 in the process, in comparison to the control sample. Only 54% of R25 was recovered, from the resin, in the case of the R25+Ca+Ale sample (Figure 6). This suggests that some of R25 remained in the solid phase, which might indicate these ions bind R25/alendronic acid tightly. This result corresponds with the previously noticed Ca-induced loss of the catalytic activity and a small response to elevation of the concentration of Mg^2+^ ions.

## 3. Materials and Methods

### 3.1. Methodology

Two ready-to-use libraries, 1 × 10^13^ PFU/mL (plaque-forming units per mL) of M13 bacteriophages with peptides displayed on the pIII protein, were chosen for the study: New England Biolabs’ PhD-C7C and PhD-12 with the architecture of N’-ACX7C-GGGS-pIII-C’ with disulfide bonds between cysteines, and N’-X12-GGGS-pIII-C’. X stands for any random proteinogenic amino acid. Since the goal of the study was to recover the catalytically active peptides, screening of the phage library was divided into two stages: affinity selection and catalytic selection.

**Figure 7:**
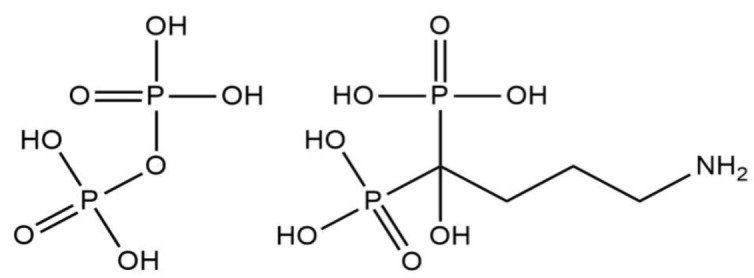
Structures of pyrophosphoric acid and alendronic acid.

**Figure 8:**
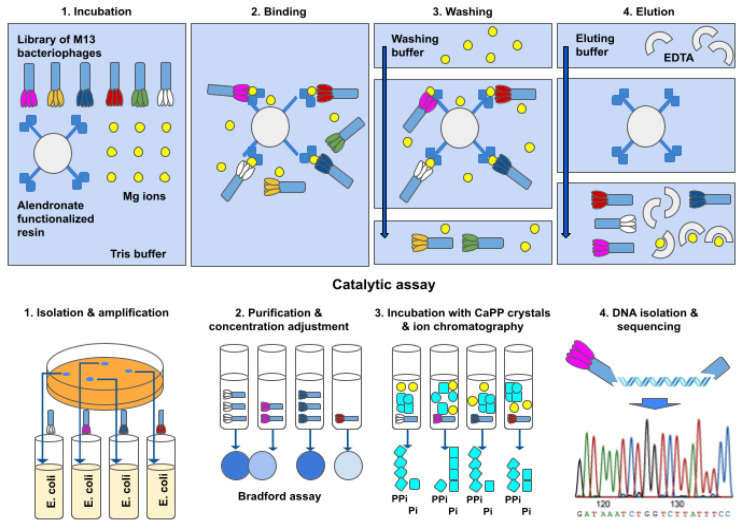
Graphical representation of bacteriophage handling and assays.

In affinity selection, the displayed peptide is expected to bind to the immobilized alendronic acid (4-amino-1-hydroxy-1-phosphonobutyl)phosphonic acid), a pyrophosphate analog (Figure 7), via the divalent cation Mg^2+^ (needed for the binding and catalysis). We chose this molecule over crystal, as the potential dissolution/hydrolysis could have happened during the binding process, resulting in a lack of affinity. Bisphosphonates such as alendronic acid do not undergo such hydrolysis. Subsequent washing with a buffer leaves only phages with a strong interaction between peptide and alendronate. The detachment was done through the introduction of a chelating agent—EDTA. Obtained this way, bacteriophages were amplified from blue colonies, leveled to the same titer, purified, and dried. The amounts of protein in samples were assessed by the Bradford method according to a BSA standard. Dissolved bacteriophages were used for catalytic selection. Then, 700 μg of bacteriophagal protein was introduced to the reaction mixture with Mg^2+^ and CaPP crystals in Tris buffer, pH 7.5. The reaction was carried out for 42 days at 50 °C to prevent any growth of possible microorganisms delivered through contamination, and relative phosphate/pyrophosphate levels in the supernatant were monitored during that period. As blind probes, we used a sample with no phages at all (-f), a sample with no phages and no Mg^2+^ (-f-M), and a sample from the host alone without the M13 phage subjected to the same method of bacteriophages isolation as all the other samples, in case any of the bacterial enzymes having hydrolytic properties. At the end of the incubation, Mg^2+^/Ca^2+^ levels in the supernatant and volume of the reaction mixture leftover were also measured. The remaining precipitate was lyophilized and the ratio of PPi to Pi amounts was measured using ion-exchange chromatography. Bacteriophages exhibiting the lowest PPi percentages were chosen for DNA isolation and sequencing. The selection process, including affinity and catalytic assays for bacteriophages, is graphically summarized in Figure 8.

Five peptides, chosen on the basis of their catalytic performance, were chemically synthesized as standalone peptides. Theu had a four amino acid-long linker, GGGS (or a one amino acid-long linker in the case of PhD-12-G3), and a 10 amino acid-long fragment of native bacteriophagal protein—AETVESSLAK—since the potential active site could have formed between native capsid protein and the displayed peptide.

Synthesized peptides were tested for CaPP hydrolysis properties along with peptide cyclo(RPDDHR) (C3) expressing some pyrophosphatase/synthetase properties [24]: serine–histidine (SH) dipeptide known from its hydrolytic abilities [25,26] and SGAGKT (W1) peptide designed to resemble a proteinaceous Walker A motif (p-loop) to bind inorganic phosphate ions [27]. Additionally, a sample with a 30 times higher concentration of Mg^2+^ (-p30xM) was prepared, as high concentrations of this ion are also known to speed up the hydrolysis of pyrophosphate [28]. The experiment was carried out for 8 days; Pi/PPi and Mg^2+^/Ca^2+^ levels were monitored during this time. The precipitate left over after the reaction was subsequently lyophilized and analyzed by ion-exchange chromatography. The final results are percentage shares of absorbance of fractions with pyrophosphate with regard to the sums of PPi and Pi absorbances.

### 3.2. CaPP Crystallization

All chemicals for this study have been supplied from Merck Life Science, Poznań, Poland, unless stated otherwise. Triclinic calcium pyrophosphate crystallization was carried out in 40 mM Tris buffer (pH 7.5, 40 mM CaCl_2_, 50 mM Na_2_P_2_O_7_·10H_2_O) over 4 months at 37 °C. It has been shown that initially some monoclinic and amorphous forms are formed during the crystallization (1 and 7% respectively) [29], so time seems to be a crucial factor, as it leads to the interconversion of all the other forms to biologically occurring triclinic form [9]. Before usage, crystals were filtered, washed with deionized water, and dried over vacuum.

### 3.3. Affinity Assay

Alendronic acid was immobilized on NovaSyn^®^ TG carboxy resin via peptide bond formation between the carboxyl residue of the resin and amine moiety of alendronic acid. First, active esters of carboxylic resin were generated in MES buffer, 50 mM, pH 6. Five equivalents of EDC were used. Subsequently, the resin was washed 3 times with the buffer, and 1.5 eq of alendronic acid in the MES buffer was added. The reaction was carried out overnight. Based on the mass difference of the dried resin before and after the reaction, the yield of the product was 92%.

Two read-to-use random libraries of M13 bacteriophages, PhD-C7C and PhD-12, 1 × 10^13^ PFU/mL were purchased from New England Biolabs (Halifax, Canada); and *E. coli* ER2738 was used as a host strain. Both bacteriophages and host bacteria strains were handled according to the producers’ manuals.

Affinity selection: 100 mg of alendronate-functionalized resin was placed into a plastic centrifuge tube filter and washed several times with the washing buffer (50 mM Tris, pH 7.5, with 50 mM concentration of Mg^2+^). To the alendronate resin dispersed in 500 μL washing buffer, 10 μL of bacteriophage library (approximately 1 × 10^11^ PFU) was added. The mixture was gently mixed and left for incubation for 20 min. Subsequently, the solution was removed by centrifugation (10 min, 2000 RPM). The unbound phages were removed by washing the resin 10 times with 500 μL of washing buffer. In the end, the detachment of phages was achieved by washing the resin twice with 600 μL of eluting buffer (50 mM Tris, pH 7.5, with 0.25 M EDTA). Since a high concentration of EDTA can destroy bacteriophages, they were precipitated. To do so, ⅙ volume of 20% PEG/2.5 M NaCl was added to the eluent, and bacteriophages were allowed to precipitate at 4 °C for 20 min. Tubes were spun at 14,000 rpm for 10 min and the supernatant was discarded. The invisible pellet was suspended in 1000 μL of TBS. Plating was done on agar/LB/IPTG/Xgal/Tet.

Bacteriophage titration of the eluent showed that the number of PFU varied from 5 to 15 from the initial 10^11^ of subjected to the affinity selection bacteriophages. The whole procedure was repeated 3 times for each library to collect the total of 47 viable bacteriophages (19 from the PhD-C7C named S01 to S19 and 28 from the PhD-12 library named R01 to R28). Three-day-old blue plaques from the bacterial lawn were stabbed with a sterile pipette tip and added to a diluted 1:100 overnight culture of a host strain in LB/Tet for amplification. After 4.5 h of incubation at 37 °C, the culture was spun 3 times for 10 min at 12,000× *g*, 4 °C, and each time the upper 80% of supernatant was transferred to a fresh tube. One sixth of the volume of 20% PEG/2.5 M NaCl was added to the remaining supernatant, and bacteriophages were allowed to precipitate at 4 °C overnight. On the next day, the tubes were spun at 14,000 rpm for 10 min and the supernatant was discarded. The invisible pellet was suspended in 100 μL of TBS. From this stock, bacteriophages were further used for catalytic selection and ssDNA isolation.

All the chemicals for the Bradford assay were bought from ThermoFisher (Schwerte, Germany) and the assay was done for the 10 μL of amplified and purified bacteriophages. The concentration assessed varied between 150 and 350 μg per sample according to the BSA standards.

### 3.4. Catalytic Assay for Bacteriophages

For the catalytic selection of bacteriophages, 700 μg of bacteriophagal protein was used. The reaction mixture consisted of 20 μL of 200 mM Tris buffer, pH 7.5; 5 μL of 100 mM of MgCl_2_; and 5 μL of CaPP crystals suspended in water. The concentration of PPi in the suspension was found by Chen’s phosphorus microdetermination method [30] to be approximately 25 mM. The final volume of the reaction mixture was 100 μL. The experiment was carried out for 42 days at 50 °C. During this time, levels of Pi/PPi were monitored. At the end of the experiment, levels of Mg^2+^/Ca^2+^ were measured [31] in the centrifuged pellet. The volume of the reaction mixture at the end of the experiment was taken into consideration while estimating concentrations of Pi/PPi and Mg^2+^/Ca^2+^. Probes that dried out due to human error or a factory defect of the Eppendorf tube were disqualified from further steps. Pellet and remnants of the reaction cocktail were used for the ion-exchange chromatography using the Bisaz et al. modified method [30] in order to determine relative quantities of PPi and Pi after the reaction.

### 3.5. ssDNA Isolation and Cloning

To isolate ssDNA, bacteriophages were lysed with 4 M NaI in TE buffer pH 8.0 for 1 h. Subsequently, ssDNA was precipitated in 70% EtOH. Before sequencing the ssDNA isolated from bacteriophages, it was amplified using PCR Mix Plus Green (A&A Biotechnology, Gdansk, Poland); forward primer 5′-TTAGTGGTACCTTTCTATTCTC-3′; reverse primer 5′-GTATGGGATTTTGCTAAACAAC-3′. Twenty-five cycles of denaturation (95 °C, 30 s), annealing (52 °C, 30 s), and elongation (72 °C, 60 s) were performed, along with 3 min of initial denaturation and 3 min of final elongation. Purification of the PCR product was performed using affinity chromatography with a commercially available GeneMatrix DNA clean-up kit (EURx, Gdańsk, Poland) in accordance with the manual. Sequencing was performed by Oligo.pl with the same primers as for amplification.

### 3.6. Peptide Synthesis

All amino acid derivatives and coupling reagents were purchased from Iris Biotech GmbH (Marktredwitz, Germany) unless stated otherwise. Peptide synthesis was performed in solid phase using the standard Fmoc strategy. Various peptides were synthesized on different types of resins depending on their sequences, the presence of disulfide bonds, and challenges in synthesis. Peptides from PhD-12 and PhD-12-3G libraries were synthesized on Wang resin. Peptides from the PhD-C7C library were synthesized on the Fmoc–Ala–Wang resin, as the direct coupling of Fmoc–Cys(Mmt)–OH to the Wang resin is not advised due to racemization—it resulted in an extension of the sequence with Ala at the C-terminus (Table 1). Extended versions of peptides with fragments of pIII protein delivered from PhD-12 and PhD-12-3G libraries and peptide W1 were synthesized on FmocLys(Boc)–Wang resin. Extended versions of peptides with cysteine bridges (library PhD-C7C) were synthesized as amides on Rink–amide resin because their synthesis in the carboxylic form on Wang resin was unsuccessful. (Table 1). Loading of the first amino acid in the case of not preloaded Wang resin was achieved in DMF with catalytic quantities of DMAP. Symmetrical anhydride of the amino acid derivative was used. The anhydride of the protected amino acid was generated in dry DCM with 0.5 equivalents (eq.) of DIC and 1 eq. of amino acid derivative. In the case of the Rink–amide resin, the first amino acid was loaded with 3 eq. PyBOP, 3 eq. 6Cl-HOBt, and 1%DIPEA. The reaction was carried out twice for 2 h. Fmoc deprotection was done in 20% piperidine in DMF. Elongation of a peptide chain was achieved with 3 eq. of amino acid derivative, 3 eq. Oxyma Pure, and 3 eq. DIC in DMF. A Kaiser test was performed before every coupling and Fmoc deprotection. Peptide C3 synthesis was described elsewhere [24]. SerHis peptide was obtained from Bachem (Bubendorf, Switzerland). Disulfide bond formation was achieved in the solid phase. First, the Mmt group was removed by treating the resin with 2% TFA in DCM. Multiple washes were performed till the color was completely washed out. Cysteine oxidation was done by treatment with 2 eq. of NCS (Merck Life Science, Poznań, Poland) in DMF for 15 min at room temperature. Full deprotection was achieved by incubation in a solution of TFA/TIS/Water 95/2.5/2.5 *v/v* for 2 h. Crude deprotected peptides were purified using preparative liquid chromatography on the c12 Jupiter Proteo column. The peak with a mass corresponding to the peptide was isolated and lyophilized. Purity was confirmed by HPLC-MS. Mass spectra were acquired on the Shimadzu LCMS-IT-TOF mass spectrometer with electrospray ionization (ESI) equipped with Jupiter Proteo column I.D. 2.0 × 250 mm, phase A: miliQ water/0.1% FA, phase B: gradient grade acetonitrile/0.1% FA, detection at 210 nm, fixed on scan mode. Elution was with linear increase from 0% to 70% of phase B in 35 min, flow = 0.2 mL/min.

### 3.7. Catalytic Assay for Peptides

For confirmation of catalytic properties of peptides, 1.8 μL of 80 mM stock peptide was used. The reaction mixture consisted of 20 μL of 200 mM Tris buffer, pH 7.5; 5 μL of 100 mM of MgCl_2_; and 5 μL of CaPP crystals suspended in water, 10% (*w/w*). The concentration of PPi in the suspension was assessed by phosphorus microdetermination to be approximately 1000 mM +/− 50 mM. The final volume of the reaction mixture was 100 μL. During the experiment, both Pi/PPi and Mg^2+^/Ca^2+^ levels in the supernatant over the centrifuged pellet were monitored. After 8 days of the experiment, the pellet and remnants of the reaction cocktail were lyophilized and used for the ion-exchange chromatography in order to determine relative quantities of PPi and Pi after the reaction.

Results of phosphate and pyrophosphate percentages in the pellet left over after the reaction were first specified as percentage shares of absorbance of pyrophosphate in elution fractions with regard to a sum of absorbances for both fractions. However, as a small part of pyrophosphate was also washed out with a fraction in the case of some phosphate, the percentage shares of absorbance for elution fractions of pyrophosphate do not coincide with the percentages of pyrophosphate in a sample. Thus, the introduction of a standard curve was necessary to assess the percentage of hydrolysis. The standard curve was created by preparing a 10% (*w/w*) suspension of calcium pyrophosphate and calcium phosphate. Subsequently, mixtures of adequate volumes of well-mixed suspensions were made to give samples corresponding to 100%, 80%, 60%, 40%, 20%, and 0% of pyrophosphate to phosphate mixes. These standard solutions were subjected to ion chromatography to assess the percentage shares of absorbance. Standard runs were subjected to ion-exchange chromatography for each round of result collection.

The determination of PPi content in a pellet left over from the reaction was achieved by using an anion-exchange resin as described by Bisaz et al., with modifications [32]. Firstly, the Dowex 1 × 8 resin was washed with 4 M HCl and subsequently with water until the pH of the eluent reached over 4. Equal amounts of resin were divided into small 2 mL columns. The swollen resin was 20 mm in height. The reaction mixtures were created after the samples were freeze-dried, dissolved in 100 μL 10% trichloroacetic acid (TCA), and applied to the column. After 15 min of incubation at room temperature, the column was washed with 1.9 mL of water. Phosphoric acid was collected while washing the resin with 0.05% HCl, and pyrophosphoric acid was collected while washing the resin with 0.5% HCl. Eight fractions of 1 mL each were collected: two after applying the sample and washing the resin with water, four after washing the resin with 0.05% HCl, and two after washing it with 0.5% HCl. The amount of Pi/PPi was determined by microdetermination of phosphorus using 50 μL of a sample.

The amounts of phosphates (both Pi and PPi) in the soluble fraction were monitored using modified Chen’s phosphorus microdetermination method [30]. For monitoring of phosphates levels during the reaction, 1 μL from the top of the centrifuged reaction mixture (or water for blind probe) was placed directly on the 96-well plate and filled up to 50 μL. Subsequently, 150 μL of a mixture of equal volumes of 6 N sulfuric acid, 0.8 M ammonium molybdate, and 10% ascorbic acid was added. After 1 h of incubation at 50 °C for the development of the color, the absorbance at 820 nm was measured against a blind probe with water. For the determination of phosphate levels in fractions after ion-exchange chromatography, 50 μL of the fraction was used. The correlation of absorbance to phosphate concentration was determined through calibration with known amounts of sodium phosphate and sodium pyrophosphate.

The determination of divalent cations was based on the reaction between o-cresolphthalein complexone (o-CPC) and Mg^2+^/Ca^2+^ in alkaline media described in [31]. First, 5 μL from the top of the centrifuged reaction mixture (or water for blind probe) was placed directly on the 96-well plate, which was topped up to 200 μL with an o-cresolphthalein complexone 0.018% (*w/v*) in 0.125 M ammonia/ammonium hydroxide buffer, pH 10.5. After 5 min of color development, the absorbance against a blind probe was measured at 570 nm. For the catalytic properties of the bacteriophages, the final values are medians from 4 separate measurements and 3 reads of absorbance each. The concentration was estimated on the basis of known concentrations of MgCl_2_. For the monitoring of Mg^2+^/Ca^2+^ levels during the confirmation of the catalytic properties of peptides, the results are medians of 3 reads of absorbance from one measurement each. The concentration was estimated on the basis of known concentrations of MgCl_2_.

All the spectrophotometric assays were performed with a NanoStar BMG spectrophotometer on a 96-well plate with at least two blind samples. A single read was a median from 16 measuring points. Before every sample was taken, reaction cocktails were spun for 4 min at 4 °C, and the required volume was taken from the top of the mixture to prevent accidental pellet intake. Before further incubation, the pellet was gently dispersed via an ultrasonic bath.

### 3.8. Molecular Dynamics

Molecular dynamics studies for R25 and R25-pIII were performed with Yasara 20.8.23, with the Amber IPQ15 force field [33], wall boundary conditions, default simulation cell size, and water molecules density 0.997 g/mL. The structure of pIII protein was taken from Protein Data Bank, accession number LG3P. During molecular dynamics, the first 10 amino acids from pIII protein were allowed to undertake the simulation along with side chains of the amino acids in the proximity of the R25 peptide. The rest of the pIII was fixed in space. We chose this fragment as it seemed like it did not form strong hydrogen bonds with the rest of the pIII protein, nor did its residues create any hydrophobic core. This is potentially could unfold and form an active site along with the displayed peptide. Additionally, fragment AETVESSLAK was the fragment we added to extended versions of peptides. Local energy minima of R25 and R25-pIII structures were calculated using the steepest descent energy minimization function.

### 3.9. R25 Affinity Assay

The affinity of R25 peptide was tested on an alendronate-functionalized resin and CaPP crystals in the presence of Mg^2+^ and Ca^2+^ ions. For this purpose, 100 mg of alendronate-functionalized resin or CaPP crystals were placed into a plastic centrifuge tube filter and washed several times with the washing buffer (20 mM Tris pH 7.5). For samples with the presence of Mg^2+^ or Ca^2+^ ions, 50 mM of the corresponding divalent cations was present in the buffer solution; then, 10 μL of R25 stock (80 mM) was added to 200 μL of proper buffer, and the mixture was incubated for 20 min. During incubation, probes were gently mixed. Afterward, the solution was removed by centrifugation (10 min, 2000 RPM). Washing was repeated two more times. Subsequently, the solid phase was washed 3 times with the eluting buffer (50 mM Tris pH 7.5, with 0.25 M EDTA). Two fractions, 600 μL each (from washing buffer and eluting buffer), were obtained from every sample. Fractions were subsequently frozen, lyophilized, and dissolved in 100 μL of miliQ water. Finally, 10 μL from each sample was analyzed via HPLC: Jupiter Proteo column; phase A: miliQ water/0.1% TFA; phase B: gradient grade acetonitrile/0.1% TFA; detection at 214 nm. Elution had a linear increase from 0% to 60% of phase B over 20 min; flow = 1 mL/min. The surface area of the washed-out R25 peptide was measured and compared to the control. The control was prepared by adding 10 μL of R25 stock solution to 100 μL of Tris buffer, pH 7.5. The surface area of this peak was considered as 100%. Each analysis was repeated 3 times.

## 4. Conclusions

Screening of random bacteriophage libraries against immobilized alendronic acid and subsequent catalytic selection led us to five peptides with potential hydrolytic properties toward CaPP crystals. Incubation of chemically synthesized peptides singled out one of them—R25—as able to lower the amount of pyrophosphate by approximately 20% in 8 days. R25’s catalysis has been shown to be Mg^2+^-dependent, and it is able to utilize soluble species of pyrophosphate with higher than 20% efficiency, which suggests that the hydrolysis is probably stopped by inhibition of calcium released from the crystal or limited access to a crystal’s surface. Similar obstacles have been met before by teams trying to use enzymatic approaches [15,16,34,35]. Previous attempts at the dissolution of CaPP crystals in vitro with chondrocyte alkaline phosphatase and pyrophosphatase resulted in a similar level of hydrolysis of PPi as shown here (the highest was 15% for chondrocyte alkaline phosphatase [36]). In vivo attempts to use enzymes, however, were unsuccessful, and this is why currently an enzymatic approach is not used in the treatment of CaPP [10,17].

Similarly to other enzymatic approaches, R25 turned out to be inhibited by Ca^2+^ ions released during the process of CaPP hydrolysis [35,37]. Since the R25 peptide is not fully washed out from the alendronate-functionalized resin, nor can it regain catalytic activity with elevated levels of Mg^2+^ after the Ca-induced inhibition, its complex with Ca^2+^ must be much stronger than the one with Mg^2+^.

Structural studies using the *in-silico* approach showed that despite strong binding of PPi and Mg^2+^, R25 lacks residues able to generate hydroxyl ions in the proximity of the central phosphorus atom the way known inorganic pyrophosphatases do [23]. Thus, the catalysis most probably is initiated by the hydroxyl ions from the alkaline solution of the reaction cocktail.

In phage display technology, it is customary to undertake two, three, or more cycles of directed evolution in order to select for more and more optimized phenotypes [38]. However, in our case, it was not possible to perform more than one round of selection. Our one selection round consisted of two stages: the first was a selection of affinity peptides from a large library of bacteriophages (ca. 6 × 10^11^); the second was a catalysis assay performed separately for each selected candidate, whose number, therefore, could not be too high. We did select the bacteriophage presenting peptide R25 for the second passage of directed evolution. However, the high titer of bacteriophages resulting from the second affinity selection (approximately 1 × 10^6^) made it impossible to screen through this number of potentially active bacteriophages in the second round of catalytic selection. Additionally, since in the first round of affinity selection we already used the strictest selection possible in order to achieve a manageable number of candidates for catalytic selection, it was not possible to further adjust the affinity pressure, resulting in 1 × 10^6^ possible second-round catalytic candidates. We learned the sequences of the peptides displayed by the two bacteriophages from the second passage. The sequences did not differ from the original R25.

Combinatorial biology approaches proved to be very effective in selecting for efficient affinity to a given molecular target. However, obtaining an efficient catalyst for a desired reaction is much more challenging. Basically, each specific reaction needs to be tackled separately, and a complex methodology needs to be established in order to obtain any positive results [39,40], and even then, the limit to the number of sequences screened is often orders of magnitudes lower than affinity selection. In our study, we designed and performed one such strategy combining the well-known combinatorial biology of phage display with a custom protocol for catalysis selection, along with chemical peptide synthesis for confirmation of the results.

Regardless of its limits, R25 might be a potential prototype for a drug against chondrocalcinosis in the future. As the directed evolution approach to enhance the abilities of R25 was too abundant in results to screen through, we believe that the continuation of this study will have to be done with rational chemical modifications. This is in fact the path that has been taken by many successful drugs in the past. For instance, the development of a peptidomimetic agent acting as an ATIC homodimerization inhibitor (for tumor growth targeting) was possible by screening peptides with a novel directed evolution strategy—SICLOPPS—by Tavassoli et al. [41], which took a decade to complete [42,43,44]. From this point of view, we are merely at the beginning of our path.

## Figures and Tables

**Table 1:** Libraries; translated sequences based on phages’ DNA; synthesized peptides and their molecular masses. Brackets indicate cyclization either via SS bridge or head to tail. Extended versions of peptides indicated with “+” in names were each synthesized with a linker (GGGS or S for R28+) and part of pIII protein (AETVESSLAK). Peptides W1 and C3 were tested as controls, since these sequences were reported to bind inorganic phosphate ions and show hydrolytic activity to PPi, respectively.

Library	Name	Translated Sequence	Synthesized Peptides	Molecular Mass [g/mol]
PhD-12-3G	R28 R28+	ADNFSGTFLFFT	H-ADNFSGTFLFFT-OH	1366.47
		ADNFSGTFLFFTSAETVESSLAK	H-ADNFSGTFLFFTSAETVESSLAK-OH	2469.65
PhD-12	R15 R15+	TTNVTEIERESY	H-TTNVTEIERESY-OH	1441.5
		TTNVTEIERESYGGGSAETVESSLAK	H-TTNVTEIERESYGGGSAETVESSLAK-OH	2715.83
PhD-12	R25 R25+	VQEDGVSLARSV	H-VQEDGVSLARSV-OH	1259.37
		VQEDGVSLARSVGGGSAETVESSLAK	H-VQEDGVSLARSVGGGSAETVESSLAK-OH	2533.7
PhD-C7C	S04 S04+	ACPSPWLGSC	H-A(CPSPWLGSC)A-OH	1089.26
		ACPSPWLGSCGGGSAETVESSLAK	H-A(CPSPWLGSC)GGGSAETVESSLAK-NH2	2291.52
PhD-C7C	S10 S10+	ACPHDSTQSC	H-A(CPHDSTQSC)A-OH	1117.17
		ACPHDSTQSCGGGSAETVESSLAK	H-A(CPHDSTQSC)GGGSAETVESSLAK-NH2	2319.45
control	W1 C3	—	H-SGAGKT-OH	519.55
		—	(RPDDHR)	776.82

## Sample Availability

Samples of the compounds tested are available from the authors.

## Abbreviations

6Cl-HOBt	6-Chloro-1-hydroxybenzotriazole dihydrate
BSA	Bovine serum albumin
CPPD	Calcium pyrophosphate deposition
CC	Chondrocalcinosis
CaPP	Calcium pyrophosphate
DIPEA	*N*,*N*-diisopropylethylamine
EDTA	Ethylenediaminetetraacetic acid
ESI	Electrospray ionization
FA	formic acid
DIC	diisopropylcarbodiimide
DMF	Dimethylformamide
DMAP	4-(Dimethylamino)pyridine
Fmoc	Fluorenylmethyloxycarbonyl protecting group
IPTG	Isopropyl-*β*-D-thiogalactoside
LB	Lysogeny broth
NCS	*N*-Chlorosuccinimide
PEG	Poly(ethylene glycol)
PFU	plaque-forming units
Pi	Phosphate
PPi	Pyrophosphate
PyBOP	Benzotriazol-1-yloxy)tripyrrolidinophosphonium hexafluorophos phate
ssDNA	single-stranded DNA
TBS	Tris buffered saline
TCA	trichloroacetic acid
Tet	Tetracycline
TFA	trifluoroacetic acid
TIS	Triisopropylsilane
TOF	Time of flight
Tris	*tris*(hydroxymethyl)aminomethane
Xgal	5-Bromo-4-chloro-3-indolyl *β*-d-galactopyranoside
